# Supratrochlear foramen of the humerus: An anatomico-radiological study with clinical implications

**DOI:** 10.1080/03009730802688819

**Published:** 2009-04-24

**Authors:** Soubhagya R Nayak, Srijit Das, Ashwin Krishnamurthy, Latha V Prabhu, Bhagath Kumar Potu

**Affiliations:** ^1^Department of Anatomy, Kasturba Medical College, CBS, BejaiMangalore, Karnataka, 575004India; ^2^Department of Anatomy, Universiti Kebangsaan MalaysiaKuala Lumpur, 50300Malaysia; ^3^Department of Anatomy, Centre for Basic Sciences, Kasturba Medical College, Manipal UniversityManipal, KarnatakaIndia

**Keywords:** Humerus, intramedullary humeral nailing, skiagram, supratrochlear foramen, translucency

## Abstract

**Background:**

The supratrochlear foramen (STF) of the humerus has been a neglected entity in standard anatomy and orthopaedics text-books. The knowledge of the presence of STF in a humerus may be important for preoperative planning for treatment of supracondylar fractures. The presence of STF may also result in erroneous interpretation of radiographs.

**Methods:**

The STF was studied in detail in 384 (220 left side and 164 right side) human dried humeri of unknown sex and age. The topographical anatomy of the STF was studied in detail, morphometric measurements were taken, and the specimens were photographed. The humerus was also taken for radiological assessment of the STF and supratrochlear septum.

**Results:**

Out of the 384 bones studied, 132 cases (34.3%) showed the presence of STF. The STF was oval, round, and triangular in shape in 123, 7, and 2 cases, respectively. The mean length of the transverse diameter for supratrochlear foramen was 6.55 mm and 5.99 mm on the left and right sides, respectively. The mean length of the vertical diameter for STF was 4.85 mm and 3.81 mm on the left and right sides, respectively. Most of the bones that had no STF showed a translucency of septum, in 56.7% of the bones.

**Conclusions:**

The results of our study show that STF is more common on the right side, with the oval shape being more common. The respective sides did not exhibit any statistical significant differences. Presence of STF may be important for anthropological, clinical, and academic purpose.

## Introduction

**Figure 1.**
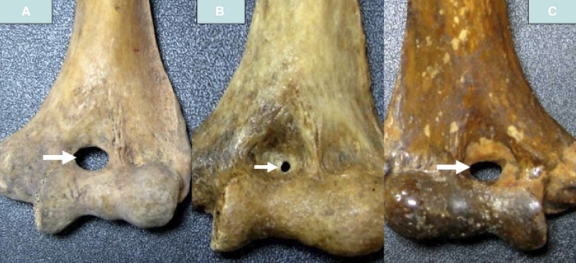
Photograph showing A: oval type of supratrochlear foramen; B: round type of supratrochlear foramen; and C: triangular type of supratrochlear foramen.

A thin plate of bone separates the olecranon and the coronoid fossa, which may become perforated in some cases to give rise to a foramen known as ‘septal aperture’ or ‘supratrochlear foramen’ (STF) (Figure 1) (1). According to Hirsh (1927) the thin plate of bone between the olecranon and coronoid fossa is always present until the age of seven years, after which the bony septum occasionally becomes absorbed to form the STF (2). Individuals with this anatomic variation may be able to overextend the elbow joint (3). Patience and a detailed look at the literature show that the STF was first described by Meckel (1825) (4). Since then, it has been described in various animals like dogs, hyenas, cattle, and other primates (5,6).

In recent times, there has been an increase in intramedullary fixation of the humerus following traumatic injuries and pathological fractures (7). One cannot forget that the supracondylar fractures are most common in the paediatric age group, and it requires proper pinning technique for stable configuration (8). The anatomical structure of the humerus may play an important role in the intramedullary fixation thereby stressing the need of prior anatomical knowledge and preoperative planning in the presence of variations like STF in the distal end of the humerus.

The humerus may be evaluated radiologically for any pathologic lesions and abnormal cysts (9,10). Prior anatomical knowledge on the variations pertaining to the supracondylar region like STF may check any erroneous interpretation of X-rays. The present anatomico-radiological study of the STF aims to highlight its incidence, morphological features, and clinical importance, which may be beneficial for anthropologists, orthopaedic surgeons, and radiologists in day-to-day clinical practice.

## Materials and methods

**Figure 2.**
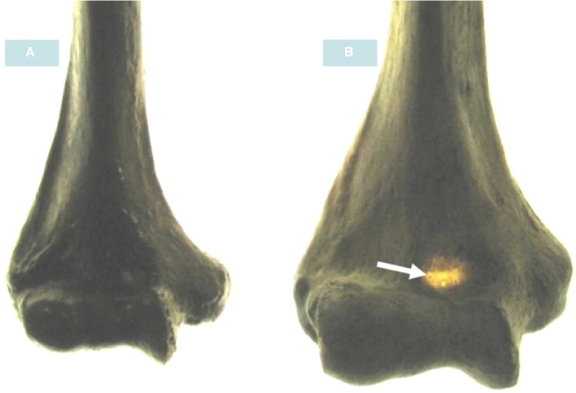
Photograph showing A: translucency of septum was absent; and B: translucency of septum was noticed.

**Figure 3.**
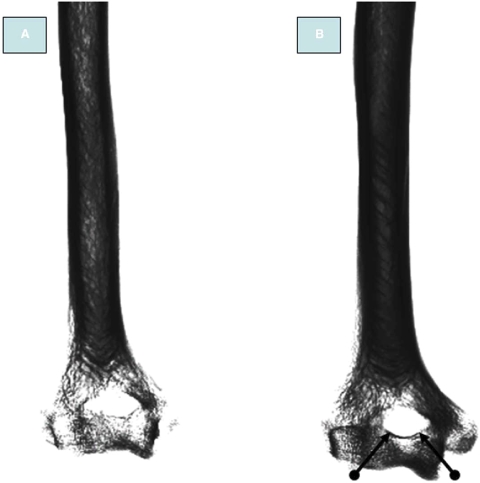
Photograph of skiagram of humerus (postero-anterior view). postero-anterior view of the lower end of the humerus. A: humerus with translucent septum; B: humerus with supratrochlear foramen. The margin of the supratrochlear foramen is clearly marked by the two arrows on the inferior aspect. Note: The translucent septum is not well margined whereas the supratrochlear foramen has clear margins.

A total of 384 (220 left side and 164 right side) human dried humeri of unknown sex and free of pathological changes, of Indian origin, were obtained from the bone bank of the Department of Anatomy, Kasturba Medical College, Mangalore, India. The presence of a STF was noted; its shape was observed and divided into three types (oval, round, and triangular). The transverse and vertical diameters of the STF and its distance from the tip of the medial epicondyle were measured using a vernier caliper. Side differences of transverse and vertical diameter of the STF were compared using the unpaired Student's *t* test; the level of significance was set at *P <* 0.05 (Table I). In bones where the foramen was absent the translucency of the septum between the coronoid and the radial fossa was noted by placing the lower end of the humerus against the X-ray lobby (Figure 2) and, to confirm that, the X-ray of the lower end of the humerus (postero-anterior view) was taken (Figure 3).

**Table I.** Different measurements in supratrochlear foramen. Data are shown as mean (standard deviation; range).

Sides	Transverse diameter for supratrochlear foramen (mm)	Vertical diameter for supratrochlear foramen (mm)
Left	6.55 (2.47; 2.3–10.3), *t*= − 1.61	4.85 (1.64; 2–7.5), *t*= − 4.50
Right	5.99 (1.47; 3.1–8.9), *t*= − 1.61	3.81 (0.97; 2.2–5.5), *t*= − 4.50

Differences between the sides were not significant (*P*>0.05, unpaired Student's *t* test).

## Results

Out of the 384 bones studied, 132 cases (34.3%) showed the presence of STF. In 59 (26.8%) cases on the left side and in 73 (44.5%) cases on the right side, a STF was present. The STF was oval, round, and triangular in shape in 123, 7, and 2 cases, respectively (Figure 1). The mean length of the transverse diameter for supratrochlear foramen was 6.55 mm and 5.99 mm on the left and right sides, respectively. The mean length of the vertical diameter for STF was 4.85 mm and 3.81 mm on the left and right sides, respectively. The differences between the sides were not significant (Table I). The incidence of STF was greater on the right side (44.5%) as compared to the left side (26.8%). The STF was absent in 252 cases (65.6%): in 161 (73.1%) cases of the left side and in 91 (55.4%) cases on the right side. Most of the bones that had no STF showed a translucency of septum (in 143 (56.7%) of the bones, in 89 (55.2%) of the left and 54 (59.3%) of the right). The nearest margin of STF was found to be commonly located at a mean distance of 26.1 mm and 28 mm on the left and right sides, respectively, from the tip of the medial epicondyle.

## Discussion

The STF, since its early description by Meckel in 1825, has been studied in many animals, but there were no anatomico-radiological studies till now. The STF had been reported in a recent anatomical study which described its incidence to be around 28% (6). There are previous studies in the Indian population which reported the incidence to be 32%, 28%, 27.5%, and 27.4% in Central Indians, South Indians, North Indians, and Eastern Indians, respectively (1,6,11,12). Our study showed an incidence of 34.4%, which is similar to earlier reports. It may be mentioned that the bones obtained in our department belonged to different individuals hailing from different parts of India, as the city of Mangalore is cosmopolitan in nature with a mixed population. The global statistics show that STF has an incidence of 4.2%, 6.9%, 7.9%, 18.1%, 21.7%, and 58% in White American, American, Egyptian, Japanese, African Negro, and Arkansas Indian populations, respectively (2,13–15). Not much data is available on the European population. The incidence of STF has ranged from 4.2% to 58% (Table II).

**Table II.** Incidence of supratrochlear foramen in various human races.

Serial no.	Author	Population studied	Incidence (%)
1	Hirsh, 1927 (quoted by Morton and Crysler)	White Americans	4.2
2	Benfer and McKern, 1966	American	6.9
3	Orztuk et al., 2000	Egyptians	7.9
4	Akabori, 1934	Ainus	8.8
5	Akabori, 1934	Japanese	18.1
6	Hirsh, 1927 (quoted by Morton and Crysler)	African Negroes	21.7
7	Chatterjee, 1968	Eastern Indians	27.4
8	Singh and Singh, 1972	North Indians	27.5
9	Singhal and Rao, 2007	South Indians	28
10	Kate and Dubey, 1970	Central Indians	32
11	Present study	Indians	34.4
12	Hirsh, 1927 (quoted by Morton and Crysler)	Arkansas Indians	58

The incidence of STF in the Indian population ranges from 28% to 34.4% (Table II). Due to the high incidence of the STF in the Indian population it requires special attention during intramedullary humeral nailing procedures in the distal portion of humerus. There is no clear opinion about the occurrence of the STF. Some authors have opined that the occurrence of the foramen is attributed to atrophy of the bone after ossification, with the impact of pressure in cases of the extension of the arm in straight-line direction (16). Animals like dog, pig, and hyena because of their posture during tearing of foods may explain the reasons for having the STF present. In human beings, the posture may not have any relation with such foramina.

The majority of the variations of the humerus focuses on the supracondylar process. A study had defined the STF to be ovoid in shape with the long axis transversal (6.3/3.7 mm) (17). The same study had advocated the compression of neurovascular structures due to the presence of such variations. In the present study, the STF was located at a mean distance of 26.1 mm and 28 mm on the left and right sides, respectively, from the tip of the medial epicondyle.

Supracondylar fractures account for 75% of all injuries in the paediatric age group (18). There is a lot of debate about the route of pin entry while treating supracondylar fractures of the humerus. The presence of the variations in the lower end of the humerus, e.g. STF, makes it more difficult to plan out such procedures preoperatively. In the present study, we observed the position of the STF to be located nearer to the medial epicondyle in the X-ray. Does that mean that the distal part of the bone cannot be used for nailing? Even intramedullary nailing cannot be possible in case these variations exist. Interestingly, a past study had observed STF in 17% cases at the fossa coronoidea, and two of them were found to have extreme antero-lateral bowing (7); this may have led to a narrow medullary canal thereby making the treatment more difficult. Thus, a STF is always associated with a narrow medullary canal in the humerus.

X-ray investigation may be performed to detect bone cysts, tumours, and other lytic conditions in day-to-day clinical practice. Prior anatomical knowledge about the presence of STF may check erroneous interpretation of X-rays by radiologists. It has been opined that the STF is an area which is relatively radiolucent, is commonly seen as a type of ‘pseudo-lesion’ in any X-ray of the upper limb, and this can be mistaken for an osteolytic or cystic lesion (3).

## Conclusion

The present study focused on the STF which is an important variation in the distal end of the humerus. To the best of our knowledge no single piece of research had performed concomitant anatomical and radiological study on the humerus while describing STF. The anatomical knowledge of STF may be beneficial for anthropologists, orthopaedic surgeons, and radiologists in day-to-day clinical practice.
